# Supplementary figures and images for: MDDeep-Ace: species-specific acetylation site prediction based on multi-domain adaptation
Source: PeerJ. 2025 Jul 3;13:e19649. doi: 10.7717/peerj.19649 (PMC12229145; doi:10.7717/peerj.19649)

# O.sativa

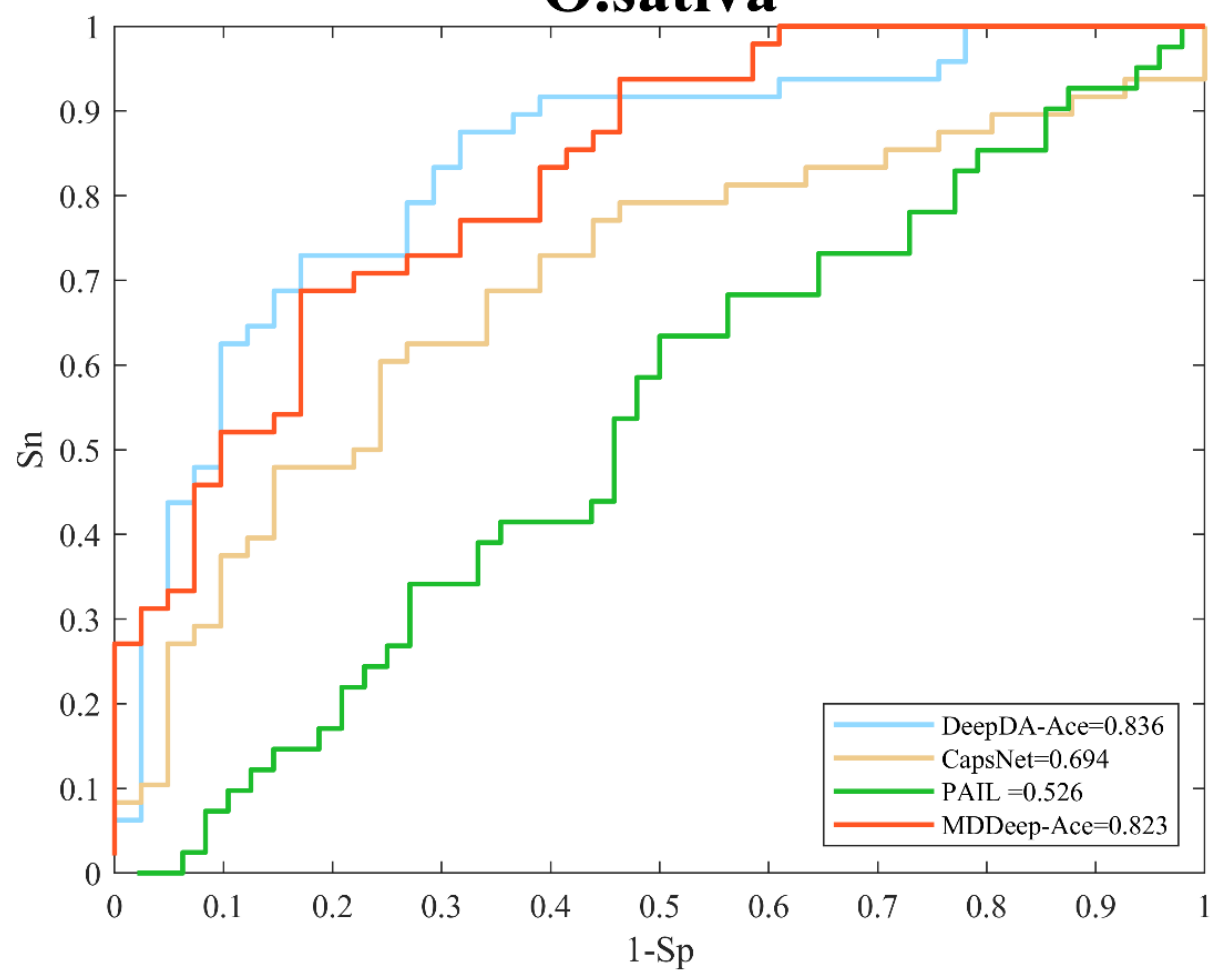

Supplement: Supplemental Information 1 [file peerj-13-19649-s001.pdf]

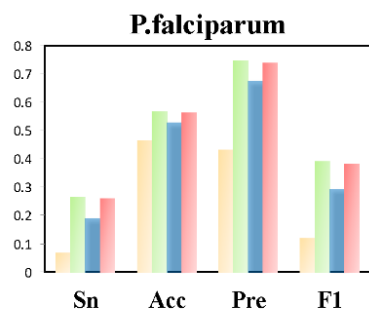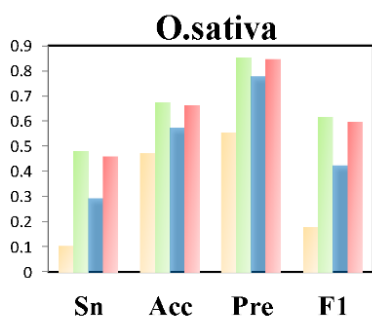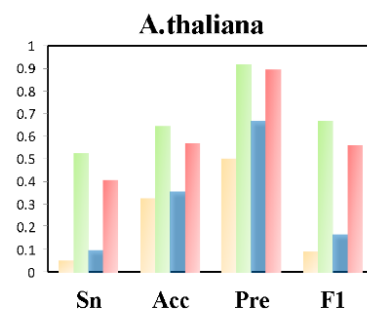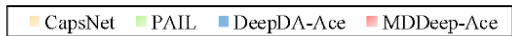

Supplement: Supplemental Information 2 — The horizontal axis represents sensitivity, accuracy, precision and F1, respectively. [file peerj-13-19649-s002.pdf]
