# Supplementary material for: MDDeep-Ace: species-specific acetylation site prediction based on multi-domain adaptation
Source: PeerJ. 2025 Jul 3;13:e19649. doi: 10.7717/peerj.19649 (PMC12229145; doi:10.7717/peerj.19649)
Supplement: Supplemental Information 3 [file peerj-13-19649-s003.docx]

Formula 1 of the paper corresponds to lines 238-267 of the code main_stage2.py.

Formula 2 of the paper corresponds to lines 315-327 of the code main_stage2.py.

Formula 3 of the paper corresponds to lines 269-278 of the code main_stage2.py.

Formula 4 of the paper corresponds to lines 280-288 of the code main_stage2.py.
