# Supplementary material for: MDDeep-Ace: species-specific acetylation site prediction based on multi-domain adaptation
Source: PeerJ. 2025 Jul 3;13:e19649. doi: 10.7717/peerj.19649 (PMC12229145; doi:10.7717/peerj.19649)
Supplement: Supplemental Information 5 [file peerj-13-19649-s005.docx]

| **Window size** | 41 | 31 | 21 |
| --- | --- | --- | --- |
| R.norvegicus | 0.767 | 0.764 | 0.756 |
| S.japonicum | 0.817 | 0.818 | 0.813 |
| S.cerevisiae | 0.808 | 0.808 | 0.801 |
| M.musculus | 0.775 | 0.772 | 0.760 |
| E.coli | 0.761 | 0.763 | 0.757 |
| B.velezensis | 0.850 | 0.847 | 0.846 |
| P.falciparum | 0.698 | 0.700 | 0.696 |
| O.sativa | 0.815 | 0.823 | 0.817 |
| A.thaliana. | 0.815 | 0.813 | 0.777 |
| Average | 0.790 | 0.790 | 0.780 |
